# Probiotic *Weizmannia coagulans* MTCC 5856 as adjunct therapy in children's acute diarrhea—a randomized, double-blind, placebo-controlled study

**DOI:** 10.3389/fped.2023.1338126

**Published:** 2024-01-10

**Authors:** Muhammed Majeed, Kalyanam Nagabhushanam, Sivakumar Arumugam, Nagarjuna Chadalavada, Jyotsna Seepana, Thumjaa Annamalai, Avinash Murali, Priji Prakasan, Lakshmi Mundkur

**Affiliations:** ^1^ Research and Development, Sami-Sabinsa Group Limited, Peenya Industrial Area, Bangalore, India; ^2^ Research and Development, Sabinsa Corporation, East Windsor, NJ, United States; ^3^ Department of Pediatrics, Aditya Multispeciality Hospital, Guntur, India; ^4^ Department of Pediatrics, Government Medical College and Government General Hospital (old RIMSGGH), Srikakulam, India; ^5^ Department of Pediatrics, Aarupadai Veedu Medical College & Hospital (AVMCH), Pondicherry, India

**Keywords:** *Weizmannia coagulans* (*Bacillus coagulans*) MTCC 5856, acute diarrhea, dehydration, oral rehydration solution, children, probiotics, randomized trial

## Abstract

**Objectives:**

Acute diarrhea in children is generally managed by replacing the lost fluid with oral rehydration solution (ORS). Probiotic supplementation has been reported to reduce the severity of diarrhea. In the present study, we investigated the effect of *Weizmannia coagulans* (*Bacillus coagulans*) MTCC 5856, along with ORS on acute diarrhea of all causes in non-hospitalized children.

**Methods:**

A total of 110 children of ages between 1 and 10 were enrolled in a double-blind placebo-controlled study and were randomly allocated to receive *W. coagulans* MTCC 5856 (4 × 10^8^ spores, *N* = 54) + ORS and zinc (Zn) or a placebo (*N* = 56) + ORS and (Zn) for 5 days. The consistency of the stool, mean duration of diarrhea in hours, mean diarrhea frequency per day, and the dehydration status were collected as efficacy endpoints. Safety was evaluated by the occurrence of adverse events.

**Results:**

The mean age of the children was 5.55 ± 2.57 years (61 boys and 49 girls). The mean duration of diarrhea was 51.31 ± 20.99 h in the *W. coagulans* MTCC 5856 group and 62.74 ± 24.51 h in the placebo (*p* = 0.011) group. The frequency of diarrhea was lower in children supplemented with the probiotic, but the difference was not statistically significant. The perceived efficacy score and dehydration status improved significantly in the *W. coagulans* MTCC 5856 group compared with the placebo group. No adverse events were recorded.

**Conclusion:**

The results of the study suggest that *W. coagulans* MTCC 5856 could be supplemented along with ORS and zinc to reduce the duration of diarrhea in non-hospitalized children.

**Clinical Trial Registration:**

ClinicalTrials.gov, identifier CTRI/2022/06/043239.

## 1 Introduction

The World Health Organization (WHO) defines diarrhea as three or more watery stools in a 24-hour period and the condition is termed as acute if the illness lasts for less than 14 days (1). Acute diarrhea is commonly due to viral and bacterial infections, and rarely parasitic infections (2, 3). As per the WHO reports, more than 1.7 billion cases of diarrhea are reported globally and more than 525,000 children under 5 years die every year, despite a mortality reduction by more than 30% between 2000 and 2015 (4, 5). Diarrhea is characterized by watery stool, increased frequency of defecation, often accompanied by fever, vomiting, and electrolyte imbalances. It is one of the main causes of dehydration, which may be life-threatening for children with malnutrition or impaired immunity (6). Oral rehydration solution (ORS), containing salts and sugar, is used as fluid and electrolytes replacement therapy for the management of acute diarrhea (1). Zinc is a vital micronutrient that gets drained during diarrhea and its supplementation along with ORS reduces the duration and severity of the illness (7, 8).

Normal bacteria in the intestine promote nutrient absorption, regulate immunity, and play an essential role in protecting the intestinal barrier. An imbalance in the gut microbiota is reported in conditions like diarrhea (9). Probiotics are microorganisms that can promote the growth of beneficial intestinal microbiota, improve intestinal environment, and promote increased immunity and resistance to pathogens (10). Recently, probiotics have gained considerable interest as they promote speedy recovery from acute diarrhea and reduce the severity of diseases (11). Several probiotic strains of *Bifidobacteria, Lactobacillus, Enterococcus faecium* SF68, *Saccharomyces boulardii*, and the combinations of different probiotics were reported to be effective in reducing the severity and duration of acute diarrhea in children (12–17). In a review of 82 clinical studies on the effects of probiotics on acute diarrhea, Collison et al. observed that the duration of hospitalization on average was shorter in probiotic groups than in control groups, although the results were highly heterogeneous (18). Recently, the European Society for Pediatric Gastroenterology, Hepatology, and Nutrition (ESPGHAN) Special Interest Group on Gut Microbiota and Modifications has recommended the use of *S. boulardii* or *L. rhamnosus* GG for the management of antibiotic associated diarrhea in children (19).

*Weizmannia coagulans* (*Bacillus coagulans*) is a Gram-positive, aerobic, or facultative anaerobic, spore-forming, lactic acid-producing bacterium (20). Several strains of *W. coagulans* are isolated from various sources and are available as probiotic supplements (21). *W. coagulans* MTCC 5856 is one such strain, identified as *generally regarded as safe* (GRAS) by the United States Food and Drug Administration for human consumption, and marketed as a dietary supplement for over three decades (22). The probiotic has excellent stability and is widely used in medicine, food, and the chemical industry (23).

*W. coagulans* MTCC 5856 showed significant antidiarrheal activities in preclinical studies (24). Furthermore, oral administration of *W. coagulans* MTCC 5856 safely improved overall gut health in diarrhea predominant irritable bowel syndrome (IBS) patients (25). Recent studies have shown that it has therapeutic effects on intestinal diseases, such as acute diarrhea, antibiotic-related diarrhea, constipation, and colitis, via modulation of the microbiota composition, host immunity, and metabolism (25–29). Additionally, toxicological experiments and many clinical observations have shown that *W. coagulans* is safe and has no effect of mutagenicity, teratogenicity, or genotoxicity (28, 30).

Some clinical studies have evaluated the safety of a few strains of *W. coagulans* in children and reported they are well tolerated (31, 32). Strain specificity is considered an important factor that determines the efficacy of probiotics in diarrhea (33). Therefore, in the present study, *W. coagulans* MTCC 5856 was supplemented along with the standard treatment of Zn and ORS to assess its safety and efficacy in children with acute diarrhea.

## 2 Methods

### 2.1 Study design

A randomized, double-blind, placebo-controlled parallel group trial was conducted in three Government centers as well as some private sectors, including Aditya Multi-Specialty Hospital, Guntur; Government Medical College and General Hospital, Srikakulam; and Aarupadai Veedu Medical College & Hospital (AVMCH), Pondicherry, from July 2022 to December 2022. The study was carried out in accordance with Good Clinical Practices as required by the International Conference on Harmonization. A written, informed consent was provided by the parents or legal guardians of all children participating in the study at the time of the child's enrolment. The study protocol (CW/97/BCG_ADRH/II/MAR/21) was approved by the institutional ethics committee (IEC) of each hospital and the trial was registered prospectively with the Clinical Trial Registry of India (CTRI) with the registration number CTRI/2022/06/043239 registered on 14/06/2022.

### 2.2 Sample size

The sample size was determined based on the baseline stool frequencies reported in an earlier publication (34). Considering the mean stool frequency value of 8.62 ± 3.17 with 80% power, alpha = 0.05, significance level and correlation of 0.4, the total sample size was determined to be 96 participants. Allowing a drop-out rate of 15%, the total sample size for recruitment was finalized as 110 children, in a 1:1 ratio between the two treatment groups (55 per treatment group).

### 2.3 Randomization and blinding

The randomization sequence table was prepared by an independent statistician using randomization software (SAS-9.4). The randomization was performed as per a computer-generated randomization schedule using block randomization method with a block size of 6.

To improve the blinding and concealment of allocation, the placebo and probiotic were coded centrally with randomization numbers as per the computer-generated randomization schedule. The participants, clinical staff, and data analysts were unaware of trial group assignments. The children enrolled in this study were randomly assigned to receive either the probiotic or the placebo. Sealed envelopes with the randomization codes were provided to the principal investigator to be opened in case of emergency.

### 2.4 Study population

Children between the ages of 1 and 10 years with clinical symptoms of acute diarrhea, as per the WHO 2005 definition, were enrolled into the study. Acute moderate diarrhea was defined as the passage of unusually loose or watery stools at least three times in the previous 24-h period, with mild or moderate signs of dehydration (the children had moderate dehydration with symptoms of restlessness and irritability, sunken eyes, and feeling thirsty, as per the WHO classification) (35, 36). The children/parents were informed about the purpose of the study by the principal investigator and the study protocol was also well explained to the parents or legal guardians who were willing to comply with all its procedures.

Exclusion criteria included the requirement for antibiotics or intravenous fluid treatment, and clinical signs of severe dehydration or severe diarrhea that requires treatment other than ORS. The children who were immunodeficient, malnourished, suffering from severe chronic illness or consuming any commercially available probiotics, or had undergone recent surgeries were also excluded from the study. In addition, children hospitalized for pneumonia, acute gastroenteritis, or having bilious vomiting, hematochezia, pancreatic dysfunction, or insufficiency as judged by medical history were also excluded from the study.

### 2.5 Intervention

The probiotic and placebo were provided by Sami-Sabinsa group of companies. Each sachet of the probiotic contained 4 × 10^8^ spores of *W. coagulans* MTCC 5856, while the placebo contained maltodextrin. Investigational products were manufactured, handled, and stored in accordance with applicable good manufacturing practice (GMP) and with the approved protocol.

The study participants were instructed to consume a one gram sachet containing *W. coagulans* MTCC 5856 (4 × 10^8^ spores) or a matching placebo dissolved in approximately 10 ml of freshly boiled and cooled drinking water twice daily along with ORS and Zn supplementation therapy. The probiotic sachets contained 0.03 g of *W. coagulans* MTCC 5856 equivalent to 4 × 10^8^ spores and 0.97 g of maltodextrin, while placebo contained 1 g of maltodextrin. The appearance of the probiotic and placebo sachets and their contents were indistinguishable. The subjects were monitored for a duration of 5 days, which involved three visits (days 1, 3, and 5) followed by telephonic contact with the parent/guardian, 3 days after the final visit to enquire about the overall well-being of the study participant. All the participants were supplied with a diary for the parent or legal guardian to complete. They were required to fill in the diary relating to study treatment, perceived efficacy scales, and stool record. Empty vials and sachets were recorded for compliance with the study.

### 2.6 Endpoints and outcome measures

The primary efficacy endpoints were the mean duration of diarrhea (expressed in hours), as counted from the time of randomization up to recovery (the first normal stool as recorded as <5 according to Bristol score as normalization of stool) and mean diarrhea frequency per day after the initiation of treatment. Frequency of stools was defined as the sum of the frequency of stools and the frequency of diapers with stools. The secondary end points were the perceived efficacy of the treatment, recorded every day by caregivers as frequency of diarrhea and dehydration status (Table 1).

**Table 1:** Perceived efficacy scale questionnaire of caregivers.

Sl. no	Frequency of diarrhea score	Dehydration score
1	More than three watery stools in a day = 4	Lethargic or unconscious = 2
2	Three watery stools in a day = 3	Not able to drink or drinking poorly = 2
3	Two watery stools in a day = 2	Sunken and dry eyes = 2
4	One watery stools in a day = 1	Skin pinch goes back very slowly (>2 s) = 2
5	Normal stool = 0	Restless and irritable = 1
6		Sunken eyes = 1
7		Drinking eagerly, thirsty = 1
8		Skin pinch goes back slowly = 1
9		No signs = 0

The questionnaire and scores are indicated in the table. The caregivers recorded their perceived efficacy and dehydration scores based on the symptoms.

The Bristol stool chart was assessed for each stool passing based on the entries made by parents in the subject diaries given to them to record diarrhea frequency and duration of diarrhea as well as investigational product compliance daily. The criteria for the evaluation of stool were explained to the parents and the children. The Bristol stool chart scale is a 7-point scale that ranges from type 1 for the hardest to type 7 for the softest stool. The stool consistency ≥5 on the Bristol stool scale is considered to be loose or liquid stool, which is indicative of diarrhea along with other symptoms (37). The caregivers were also asked to assess the signs of dehydration including pinch analysis on abdomen skin and were instructed to record their score on the perceived efficacy scale questionnaire. As an exploratory end point, Rota virus and Adenovirus were screened at baseline to evaluate the efficacy of the study treatment in children with confirmed viral diarrhea.

### 2.7 Safety

The study participants were monitored for safety data throughout the course of the study. All the children underwent general physical examination including recording of vitals such as temperature, pulse, and respiratory rate at baseline and on day 5. The occurrences of any adverse events and/or serious adverse events were monitored throughout the study period.

### 2.8 Statistical analysis

The normality of the quantitative variables was analyzed using the Shapiro–Wilks test, and the data are represented as means, standard deviation (SD), or median, and range based on their distribution. The differences within the group at different time points were compared by repeated measures analysis of variance (ANOVA) and Dunnett's multiple comparison. For categorical variables, the frequency and percentage of the population were presented. A descriptive comparison was provided to differentiate the treatment effect between the treatment groups. For continuous variables, the Mann–Whitney *U* test was performed for the comparative analysis between treatment groups for non-normally distributed data and the results were presented as mean and *p*-value. Comparative analysis was performed using the Wilcoxon signed rank test for the non-normally distributed data within the group and the results were presented as mean and *p*-value. All the statistical analysis was performed by STATA Software version 16.0 by an independent statistician blinded to the study groups. The level of significance was defined as a *p*-value less than 0.05.

## 3 Results

### 3.1 Demographic characteristics

**Figure 1:**
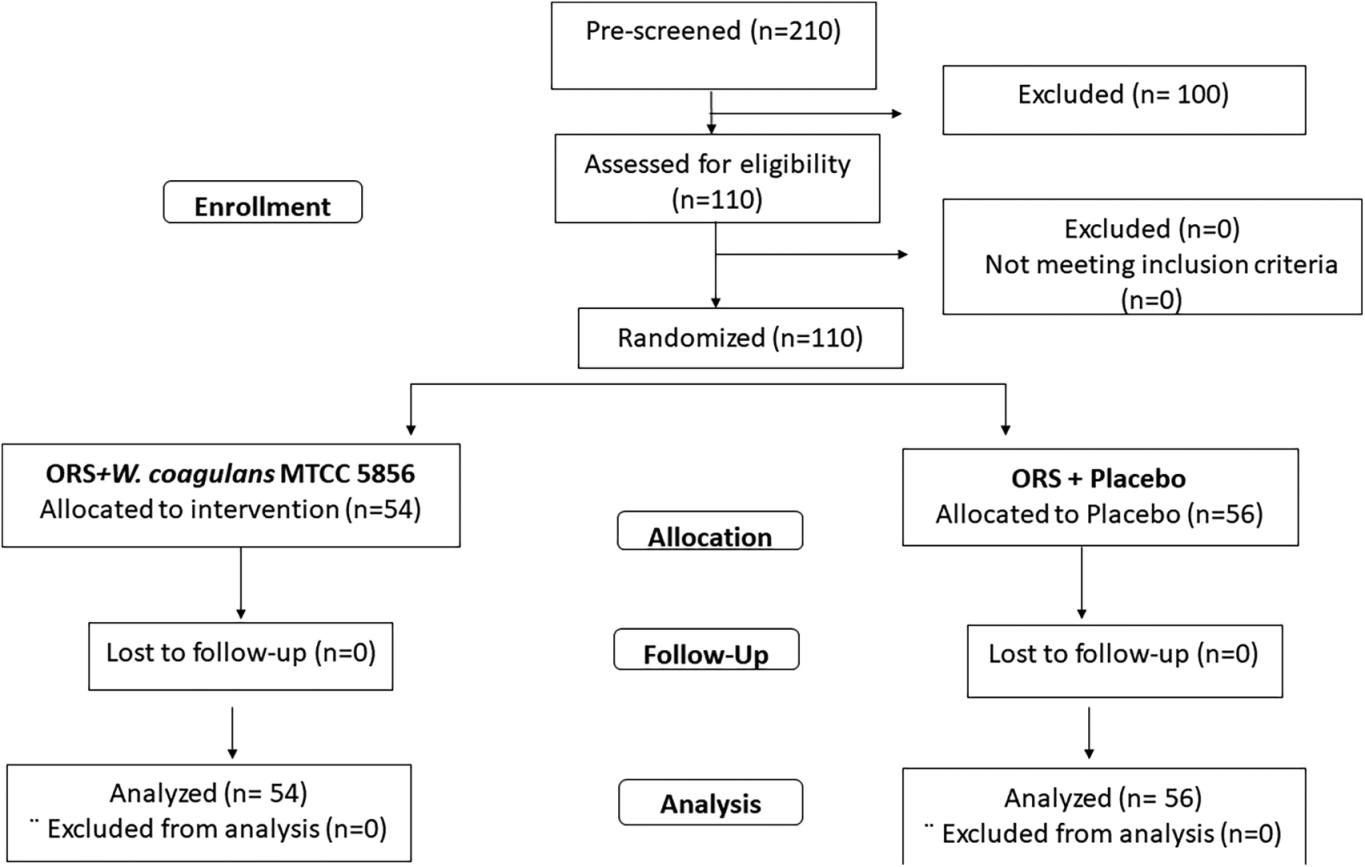
Consort diagram. Patient disposition is represented in the consort diagram.

A total of 210 children diagnosed with acute diarrhea were prescreened and 110 children, who met the inclusion criteria, were randomized to receive *W. coagulans* MTCC 5856 (*n* = 54) or a placebo (*n* = 56) (Figure 1). The baseline demographic characteristics were well balanced between the two groups. The mean age of the children was 5.55 ± 2.57 years (5.78 ± 2.76 in the probiotic and 5.34 ± 2.38 in the placebo groups) and the mean weight was 21.44 ± 8.34 kg (22.19 ± 8.41and 20.71 ± 8.28) with an average BMI of 17.83 ± 8.37 (18.89 ± 11.36 and 16.80 ± 3.52) in *W. coagulans* MTCC 5856 and placebo, respectively. Among the study participants, 55.45% were boys and 44.54% were girls. A total of 34 (30.90%) children were positive for adenoviruses in stool (18 in the probiotic group and 16 in the placebo group). Rotavirus was found in the stools of 23 (20.90%) children; 10 in the active group and 13 in the placebo group, while 9 children were positive for both viruses (5 in the probiotic group and 4 in the placebo group). The details of the demographic characteristics are presented in Table 2.

**Table 2:** Demographic characteristics of study participants.

Parameter	Active (*N* = 54)	Placebo (*N* = 56)	Overall (*N* = 110)
Age (years)	5.78 ± 2.76	5.34 ± 2.38	5.55 ± 2.57
Gender, *n* (%)	** **	** **	** **
Male	30 (55.56%)	31 (55.36%)	61 (55.45%)
Female	24 (44.44%)	25 (44.64%)	49 (44.54%)
Height (cm)	110.41 ± 17.52	108.84 ± 16.54	109.62 ± 16.97
Weight (kg)	22.19 ± 8.41	20.71 ± 8.28	21.44 ± 8.34
BMI (kg/m^2^)	18.89 ± 11.36	16.80 ± 3.52	17.83 ± 8.37
Race, *n* (%)	** **	** **	** **
Asian	54 (100.00%)	56 (100.00%)	110 (100.00%)
Rotavirus			
Positive	10 (18.51%)	13 (23.21%)	23 (20.90%)
Negative	44 (81.48%)	43 (76.78%)	87 (79.09%)
Adenovirus			
Positive	18 (33.33%)	16 (28.57%)	34 (30.90%)
Negative	36 (66.67%)	40 (71.42%)	76 (69.09%)
Both positive	5 (9.25%)	4 (7.14%)	9 (8.18%)

The values are given as mean and standard deviation except for gender, race, and viral screening, which are represented as number of children and percentage. BMI, body mass index.

### 3.2 Duration and frequency of diarrhea

**Figure 2:**
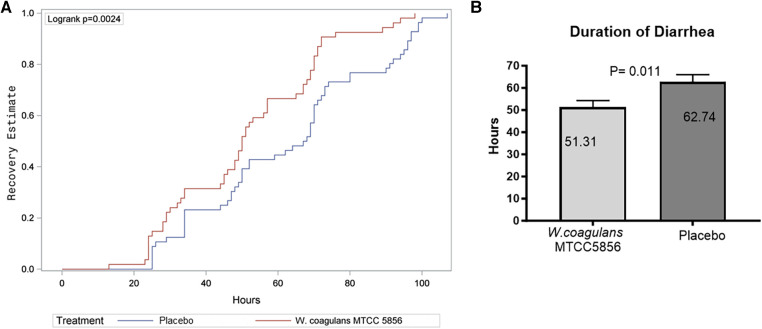
Recovery from diarrhea. (**A**) Kaplan–Meier estimate of recovery from diarrhea. (**B**) Duration of recovery in placebo and *W. coagulans* MTCC5856. Recovery was defined as the first normal stool recorded according to Bristol score; a score <5.

The primary outcome parameters were the analysis of mean duration and frequency of diarrhea from randomization to recovery. The children in the probiotic group showed a mean recovery (normal stool with Bristol stool chart score <5) after 51.43 ± 20.76 h, whereas the children in the placebo group showed recovery after 62.74 ± 24.51 h of treatment, which was significantly better (*p* = 0.012) in the probiotic supplemented children (Figure 2A). The median recovery and confidence interval (CI) were 50.43 (45.76, 57.09) h for the *W. coagulans* MTCC 5856 group and 67.65 (56.17, 69.30) h for the placebo group. The net reduction in diarrhea duration was 17.22 h (95% CI: 1.3–21.40, *p* = 0.012), calculated as 67.65–50.43 h. A Kaplan–Meier curve, generated to compare the total duration of diarrhea between groups, showed a statistically significant difference between treatment groups with a log rank *p*-value of 0.0024 and hazard ratio of 0.55 (95% CI: 0.37, 0.82) (Figure 2B). In the probiotic supplemented group, 53/54 (98.14%) children recovered from diarrhea in 5 days, while 50/56 (89.28%) children recovered in the placebo group. The cumulative recovery was better in the *W. coagulans* MTCC 5856 group compared with the placebo group.

The mean frequency of diarrhea per day did not differ significantly between groups. A gradual decrease in the frequency was observed in both treatment groups. (Table 3). Repeated measure ANOVA within the group showed a significant improvement in both *W. coagulans* and the placebo groups from day 3 onward.

**Table 3:** Frequency of diarrhea.

Parameter	Active (*N* = 54)	Placebo (*N* = 56)	*p*-Value
Frequency of diarrhea in first 6 h			
1	3 (5.56%)	0 (0.00%)	0.0737
2	16 (29.62%)	17 (30.35%)	0.9337
3	30 (55.56%)	31 (55.35%)	0.9833
4	5 (9.25%)	6 (10.71%)	0.7993
5	0 (0.00%)	2 (3.57%)	0.1611
Mean frequency	2.69 ± 0.72	2.88 ± 0.74	0.33
Frequency of diarrhea on Day 2			
0	2 (3.70%)	0 (0.00%)	0.1461
1	1 (1.85%)	0 (0.00%)	0.3063
2	15 (27.78%)	8 (14.28%)	0.0819
3	22 (40.74%)	33 (58.92%)	0.0565
4	14 (25.92%)	15 (26.78%)	0.9185
Mean frequency	2.83 ± 0.97	3.13 ± 0.63	0.13
Frequency of diarrhea on day 3			
0	8 (14.81%)	12 (21.42%)	0.3686
1	14 (25.92%)	3 (5.35%)	0.0028
2	8 (14.81%)	14 (25.00%)	0.1819
3	12 (22.22%)	17 (30.35%)	0.2808
4	12 (22.22%)	10 (17.85%)	0.6343
Mean frequency	2.11 ± 1.41	2.18 ± 1.39	0.82
Frequency of diarrhea on day 4			
0	14 (25.92%)	13 (23.21%)	0.8252
1	13 (24.07%)	5 (8.92%)	0.0318
2	8 (14.81%)	17 (30.35%)	0.0518
3	8 (14.81%)	10 (17.85%)	0.6663
4	11 (20.37%)	11 (19.64%)	0.9240
Mean frequency	1.80 ± 1.50	2.02 ± 1.42	0.42
Frequency of diarrhea on day 5			
0	25 (46.29%)	19 (33.92%)	0.1856
1	18 (33.33%)	22 (39.28%)	0.5165
2	10 (18.51%)	9 (16.07%)	0.7343
3	1 (1.85%)	6 (10.71%)	0.0570
Mean frequency	0.76 ± 0.81	1.04 ± 0.97	0.15

Values are represented as Number (percentage). The mean frequency and standard deviation are given for each day. The improvement within the two groups was computed using repeated measure ANOVA and was found to be significant for both placebo and *W. coagulans*.

### 3.3 Dehydration score and perceived efficacy by caregivers

The difference between the dehydration status was evaluated as per WHO classification. The dehydration status improved (reduction in score) in all children with time and was found to be statistically significant in children supplemented with *W. coagulans* MTCC 5856 (2.37 ± 1.56 on day 1–0.00 on day 5) compared with the placebo (2.16 ± 1.58 to 0.09 ± 0.29, *p* = 0.026 on day 5) (Table 4). The perceived efficacy score as recorded by the caregivers also improved in both groups and was significantly better in the probiotic group on day 5 (3.69 ± 0.75 to 0.00 in the probiotic group and 3.73 ± 0.59 to 0.23 ± 0.54 in the placebo group, *p* < 0.001).

**Table 4:** Dehydration and perceived efficacy.

	Active (*N* = 54)	Placebo (*N* = 56)	Inter group *p*-value
Dehydration score			
Day 1	2.37 ± 1.56	2.16 ± 1.58	0.47
Day 3	0.83 ± 0.80	0.96 ± 1.19	0.92
Day 5	00 ± 00	0.09 ± 0.29	0.026*
Perceived efficacy score			
Day 1	3.69 ± 0.75	3.73 ± 0.59	0.96
Day 3	1.67 ± 1.18	1.93 ± 1.19	0.23
Day 5	0.00 ± 0.00	0.23 ± 0.54	0.001**

The dehydration status was evaluated as per WHO classification. The perceived efficacy of the treatment was recorded by caregivers based on the symptoms. A lower score indicates a better outcome. Values are represented as mean ± SD.

* *p* < 0.05.

** *p* < 0.005.

### 3.4 Duration of recovery in viral diarrhea

The children who were positive for rotavirus or adenovirus at baseline took a longer duration for recovery in both groups, although the difference did not reach statistical significance. The median duration of diarrhea was shorter in the *W. coagulans* MTCC 5856 supplemented group (59.95 ± 18.88 h in rotavirus and 58.82 ± 21.42 h in adenovirus positive children) compared with the placebo (70.11 ± 17.57 h and 62.86 ± 26.89 h in rotavirus and adenovirus positive children, respectively), but the difference was not significant. The median recovery and confidence interval were 48.50 (43.15, 55.84) h for the *W. coagulans* MTCC 5856 group and 62.00 (52.50, 68.51) h for the placebo group. The net reduction in diarrhea duration was 13.50 h (95% CI: 0.2–22.30, *p* = 0.03), calculated as 62.0–48.50 h in rotavirus negative children. The median recovery and confidence interval in adenovirus negative children was 48.50 (41.07, 54.39) h for the *W. coagulans* MTCC 5856 group and 66.50 (54.26, 71.46) h for the placebo group. The net reduction in diarrhea duration was 18.00 h (95% CI: 1.9–26.60, *p* = 0.007), calculated as 66.50–48.50 h. Probiotic supplementation reduces the recovery time in children positive for the virus, but it was not significant most likely due to the small numbers in the present study (Table 5).

**Table 5:** Duration of recovery in viral diarrhea.

Category	Duration of diarrhea (h)		*p*-Value between the groups
	*W. coagulans* MTCC 5856	Placebo	
Rota virus positive	*N* = 10	*N* = 13	0.21
	59.95 ± 18.88	70.11 ± 17.57	
Rota virus negative	*N* = 44	*N* = 43	0.037*
	49.49 ± 20.87	60.51 ± 26.01	
Adeno virus positive	*N* = 18	*N* = 16	0.57
	58.82 ± 21.42	62.86 ± 26.89	
Adeno virus negative	*N* = 36	*N* = 40	0.007*
	47.73 ± 19.68	62.43 ± 17.94	

Values are represented as mean ± SD.

* *p* < 0.05.

### 3.5 Safety

Vital signs were also measured as a part of the safety analysis, and no abnormal or out-of-range values were observed. Further, no changes associated with the treatment in safety measurements including adverse events and/or serious adverse events were reported in both groups (Table 6). The vital signs like diastolic and systolic blood pressures (67.28 ± 7.95 /102.15 ± 10.90 and 70.21 ± 9.05/ 102.77 ± 10.82), pulse rate (94.80 ± 9.68 and 95.29 ± 9.69), and body temperature (97.99 ± 0.73 and 98.17 ± 0.58) in the probiotic and placebo groups, respectively, were normal in both groups at the end of the study (Table 7).

**Table 6:** Safety and adverse events.

Group	AE	Outcome	Severity	Possible relationship to study drug	Action taken	Withdrawn due to AE	Serious AE (SAE)
Placebo	Gastric irritation	Resolved	Mild	No	Treatment continued	No	No
*W. coagulans*	Gastric irritation	Resolved	Mild	No	Treatment continued	No	No
Placebo	Gastric irritation	Resolved	Mild	No	Treatment continued	No	No
Placebo	Gastric irritation	Resolved	Mild	No	Treatment continued	No	No

Minor adverse events like gastric irritation were recorded, which were resolved and treatment continued for all children.

**Table 7:** Summary of vital signs.

Parameter	Active (*N* = 54)		Placebo (*N* = 56)		*p*-Value
	Day 1	Day 5	Day 1	Day 5	
Systolic blood pressure (mmHg)	102.37 ± 10.91	102.15 ± 10.90	102.77 ± 10.82	102.05 ± 11.02	0.4933
Diastolic blood pressure (mmHg)	66.70 ± 9.00	67.28 ± 7.95	70.21 ± 9.05	69.50 ± 6.30	0.1017
Body temperature (°F)	98.03 ± 0.89	97.99 ± 0.73	98.25 ± 1.10	98.17 ± 0.58	0.2285
Pulse rate (beats/min)	97.24 ± 12.42	94.80 ± 9.68	98.11 ± 11.52	95.29 ± 9.69	0.8811

Values are represented as mean ± SD.

## 4 Discussion

In the present study, we compared the efficacy of ORS and zinc therapy with probiotic supplementation against acute diarrhea in children. *W. coagulans* MTCC 5856 +ORS, zinc supplementation resulted in significantly faster recovery from diarrhea in children in the age group of 2–10 years. The perceived efficacy score and dehydration improvements were better with the addition of probiotics compared with the zinc ORS therapy. The frequency of diarrheal episodes decreased significantly in both groups from day 3 onward. We could not observe any differential effect of probiotic in viral diarrhea, which could be due to the low number of positive cases in our study.

The WHO recommends treating diarrhea with ORS containing sugar and salt in clean water with 20 mg zinc to improve the outcomes (35). Oral hydration therapy with ORS and Zn supplementation is reported to have beneficial effects on the absorption of water and electrolytes by the intestine, regeneration of gut epithelium, and increased levels of enterocyte brush-border enzymes (38). Clinical evidence has shown that reducing the duration of diarrhea is crucial in reducing the risks of severe dehydration in children (39, 40).

Probiotics have also been introduced as adjuvant therapy for the management of diarrhea since the last few decades. However, clinical studies investigating the efficacy of probiotics for the treatment of diarrheal diseases reported contentious results with both efficacious and not-so-positive effects (14, 18). Probiotic treatment was reported to significantly reduce the duration of diarrhea in children with mild diarrhea in earlier studies (16, 41). Children with acute gastroenteritis were reported to benefit with supplementation with probiotic strains like *L. reuteri* ATCC 55730, *L. rhamnosus* 19070-2, and *L. reuteri* DSM 12246 (17, 42). A recent study in children with infectious diarrhea claimed that inclusion of a probiotic mixture was safe and effective in shortening the duration of watery diarrhea and faster discharge form hospital (43). In an earlier study*, Lactobacillus sporogenes* (*B. coagulans*) was reported to show no therapeutic impact on management of acute diarrhea as an adjunct to ORS (31). A meta-analysis suggested that the duration of diarrhea was shorter in the probiotic group and the supplementation with combination of probiotics had a better effect than a single probiotic (44). Supplementation of the probiotic bacteria such as *Lactobacillus reuteri*, *Saccharomyces boulardii*, *Lactobacillus rhamnosus,* and *Lactobacillus acidophilus* increased the treatment efficacy and reduced the duration of hospital stay (45–47). In contrast to these studies, *L. sporogenes* (*B. coagulans*) was reported to show no therapeutic impact on management of acute diarrhea as an adjunct to ORS (31). Similarly, supplementation with *B.clausii* and ORS + zinc showed no significant improvement in acute diarrhea in infants (48). In corroboration with some of the studies with probiotics, we observed a reduction in the duration of diarrhea, by the addition of *W. coagulans* MTCC 5856 to ORS and zinc mixture.

The mechanism of action of *W. coagulans* MTCC 5856 in acute diarrhea is not fully understood. However, it has been shown that *W. coagulans* can interact with several pathways to produce beneficial effects. *W. coagulans* MTCC 5856 is known to possess a broad spectrum of antimicrobial activity, which may help to maintain microbial homeostasis and resist the growth of pathogens. In addition, the strain produces organic acid (lactic acid) and short-chain fatty acids (SCFAs), which may lower the pH of the gut cavity, thereby inhibiting the growth of pathogens. These short-chain fatty acids play a pivotal role in the maintenance of overall gut health, intestinal morphology, and function (27, 30, 49). Moreover, *W. coagulans* MTCC 5856 is reported to have immunomodulatory properties in human colonic cells, which make the spores efficacious in the management of infectious diarrhea (50). *In vivo* studies also reported that *W. coagulans* MTCC 5856 increased the expression of tight junction proteins and reduced the damage caused by inflammation in gut tissues (51). *W. coagulans* MTCC 5856 also elicited antidiarrheal activity and inhibited the gastrointestinal motility in fasted rats (24). Similar effects were translated to clinical studies also where *W. coagulans* MTCC 5856 significantly reduced the diarrhea, abdominal pain, and stool frequency in diarrhea predominant IBS patients (25).

Stringent quality control and safety procedures are mandatory for probiotics, when considering the vulnerability of pediatric population. Supplementation with *W. coagulans* MTCC 5856 was well tolerated and there were no unexpected adverse events during the study. Minor adverse events reported were similar between the placebo and the probiotic groups. The present study reported no significant changes in the vital signs and no adverse effects were reported by any of the participating children, reiterating the safety of *W. coagulans* MTCC 5856.

The major limitation of the study was the relatively smaller number of recruited children. All the children were recruited from the southern part of India, restricting the ethnic diversity. We did not analyze the effect of the probiotic on gut microbiome, which could have added value to the mechanism of action of the strain. However, the study was well designed and conducted as a double-blind, placebo-controlled trial, comparing ORS with zinc treatment with the same enriched with *W. coagulans* MTCC 5856 in children with acute diarrhea.

## 5 Conclusion

The present randomized, double-blind, placebo-controlled trial showed that *W. coagulans* MTCC 5856 supplemented with ORS and Zn had comparatively more efficacy than ORS and Zn treatment alone in managing acute diarrhea in children without malnutrition. Further, this is the first time the specific strain of *W. coagulans* MTCC 5856 is being tested for the safety and efficacy in children with acute diarrhea, which make the results clinically important for health professionals, including general practitioners, general pediatricians, pediatric gastroenterologists, and nutritionists.

## Data Availability

The original contributions presented in the study are included in the article/Supplementary Material, further inquiries can be directed to the corresponding author.
